# Supplementary material for: Initiation of Translation by Cricket Paralysis Virus IRES Requires Its Translocation in the Ribosome
Source: Cell. 2014 May 8;157(4):823–31. doi: 10.1016/j.cell.2014.04.015 (PMC4017093; doi:10.1016/j.cell.2014.04.015)
Supplement: Table S1. Refinement and Model Statistics for the Final Refined Models, Related to Figure 1 [file mmc1.pdf]

Table S1. Refinement and Model Statistics for the Final Refined Models, Related to Figure 1

**Data Collection**	**40S/IRES**	**60S**
Particles	25,969	18,132
Pixel size (Å)	1.34	1.34
Defocus range (µm)	1.8-3.5	1.8-3.5
Voltage (kV)	300	300
Electron dose (e- nm^{-2})	25	25
**Model composition**		
Non-hydrogen atoms	79,660	125,688
Protein residues	4,828	6,450
RNA bases	1,781	3,483
**Refinement**		
Resolution used for refinement (Å)	4.0	3.8
Map sharpening B-factor ($Å^2$)	-107.6	-107.6
Average B factor ($Å^2$)	144.7	124.3
R factor†	0.27	0.29
Fourier Shell Correlation (FSC)*	0.79	0.85
**Rms deviations**		
Bonds (Å)	0.0081	0.0042
Angles (°)	2.68	1.84
**Ramachandran plot**		
Favored (%)	76.02	83.13
Outliers (%)	7.8	5.52

† R factor = $\Sigma||F_{obs}|-||F_{calc}| / \Sigma|F_{obs}|$

* FSC=$\Sigma(F_{obs}F^{*}_{calc}) / \sqrt{(\Sigma|F_{obs}|^2 \Sigma|F_{calc}|^2)}$
