# Supplementary material for: Initiation of Translation by Cricket Paralysis Virus IRES Requires Its Translocation in the Ribosome
Source: Cell. 2014 May 8;157(4):823–31. doi: 10.1016/j.cell.2014.04.015 (PMC4017093; doi:10.1016/j.cell.2014.04.015)
Supplement: Document S1. Article plus Supplemental Information [file mmc2.pdf]

Cell

Initiation of Translation by Cricket Paralysis Virus IRES Requires Its Translocation in the Ribosome

Israel S. Fernández,[1] Xiao-Chen Bai,[1] Garib Murshudov,[1] Sjors H.W. Scheres,[1] and V. Ramakrishnan[1,*]
[1]MRC Laboratory of Molecular Biology, Francis Crick Avenue, Cambridge Biomedical Campus, Cambridge CB2 0QH, UK
*Correspondence: ramak@mrc-lmb.cam.ac.uk
http://dx.doi.org/10.1016/j.cell.2014.04.015
This is an open access article under the CC BY license (http://creativecommons.org/licenses/by/3.0/).

SUMMARY

The cricket paralysis virus internal ribosome entry site (CrPV-IRES) is a folded structure in a viral mRNA that allows initiation of translation in the absence of any host initiation factors. By using recent advances in single-particle electron cryomicroscopy, we have solved the structure of CrPV-IRES bound to the ribosome of the yeast *Kluyveromyces lactis* in both the canonical and rotated states at overall resolutions of 3.7 and 3.8 Å, respectively. In both states, the pseudoknot PKI of the CrPV-IRES mimics a tRNA/mRNA interaction in the decoding center of the A site of the 40S ribosomal subunit. The structure and accompanying factor-binding data show that CrPV-IRES binding mimics a pretranslocation rather than initiation state of the ribosome. Translocation of the IRES by elongation factor 2 (eEF2) is required to bring the first codon of the mRNA into the A site and to allow the start of translation.

INTRODUCTION

Initiation of protein synthesis requires the accurate positioning of the initiator aminoacyl-tRNA and the start codon of the messenger RNA (mRNA) in the ribosomal P site. Whereas bacterial initiation requires just three initiation factors and generally a short Shine-Dalgarno sequence near the 5′ end of mRNA, eukaryotic initiation is far more complex, requiring almost a dozen initiation factors or eIFs (reviewed by Marintchev and Wagner, 2004). Moreover, it is becoming increasingly clear that much translational control of gene expression occurs through regulation of initiation.

In eukaryotes, mRNAs are capped at the 5′ end by 7-methylguanosine (reviewed in Jackson et al., 2010; Aitken and Lorsch, 2012). A preinitiation complex of the 40S ribosomal subunit with eIFs 1, 1A, 3, and 5, along with the ternary complex of eIF2, guanosine triphosphate (GTP), and initiator tRNA (Met-tRNA$_i^{Met}$), is recruited to the 5′ end of mRNA via the eIF4 complex. Intensive biochemical and genetic studies have established the dynamic nature of the start codon recognition in eukaryotes, involving a "scanning" mechanism that eventually results in the initiator aminoacyl tRNA being correctly base paired with the start codon at the P site. Finally, eIF5B, the eukaryotic ortholog of the bacterial protein IF2, assists in the recruitment of the large subunit.

Many viruses circumvent host control of translational initiation by dispensing with some or all of these cellular initiation factors through special sequences on their mRNA referred to as internal ribosomal entry sites (IRES) (Filbin and Kieft, 2009). Such IRES sequences, which can be located far from the 5′ end of mRNA and sometimes in the intergenic region of a polycistronic message, can be classified accordingly to their dependency on canonical initiation factors for translation. At one extreme are the class IV IRES sequences, which enable ribosome to translate their messages independently of any cellular initiation factors, exemplified by the widely characterized cricket paralysis virus IRES (CrPV-IRES) (Wilson et al., 2000). It was shown that CrPV-IRES, which occurs in an intergenic region of a dicistronic message, binds first to the 40S, then recruits the large subunit to directly initiate synthesis of the downstream gene from the A site of the ribosome rather than the P site as in canonical initiation (Wilson et al., 2000). CrPV-IRES was also shown to initiate translation in the yeast *Saccharomyces cervisiae* (Thompson et al., 2001), showing that it can function in widely divergent species.

The CrPV-IRES sequence consists of ∼190 nucleotides that fold into three internal pseudoknots (termed PKI, II, and III; Figure 1A) (Kanamori and Nakashima, 2001). The PKI pseudoknot of the CrPV-IRES is thought to mimic the initiator tRNA/mRNA interaction and thus to establish the correct reading frame in the viral messenger upon interaction with the ribosome (Costantino et al., 2008). Previous low-resolution cryo-EM reconstructions showed that the CrPV-IRES was localized in the intersubunit space of the ribosome, in approximately the same region in which the tRNAs and the mRNA interact with the ribosome (Spahn et al., 2004). High-resolution structures of isolated domains of CrPV-IRES (Pfingsten et al., 2006) (Costantino et al., 2008), as well as a more recent cryoEM study (Schüler et al., 2006), have shed further light on its structure. However, a high-resolution structure of the entire molecule in the context of the ribosome will greatly facilitate our understanding of CrPV-IRES function, including how it sets the proper reading frame in the ribosome and facilitates the first translocation event in the absence of peptide bond formation.

Recent advances in electron cryomicroscopy (cryo-EM) now make it possible to obtain high-resolution structures of

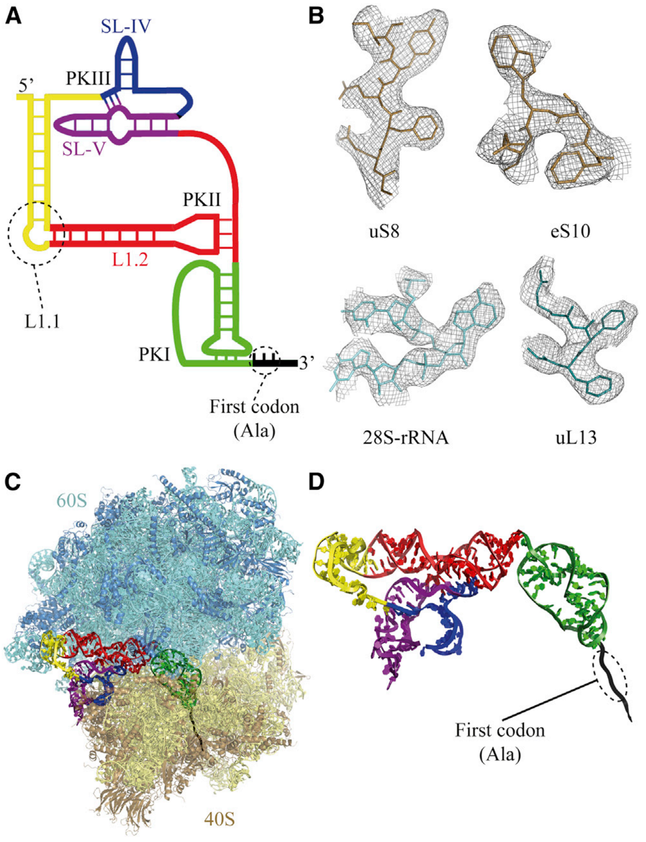

Figure 1. Structure of CrPV-IRES in the Ribosome

(A) Secondary structure of the CrPV-IRES RNA.

(B) Density of cryoEM maps used to build the structure of CrPV-IRES bound to the ribosome in the rotated state.

(C) An atomic model of CrPV-IRES bound to the ribosome of the yeast *K. lactis*.

(D) Tertiary structure of the CrPV-IRES in the ribosome, color coded according to the secondary structure diagram in (A).

ribosomes from a relatively small number of particles (Bai et al., 2013; Li et al., 2013). Accordingly, we have used cryo-EM to determine the structures of several conformations of the CrPV-IRES/ribosome complex at resolutions beyond 4 Å, which allowed us to build and refine complete atomic models for the complex. These structures reveal both the conformational variation of the IRES in the ribosome as well as unexpected insights into how the CrPV-IRES initiates translation.

RESULTS

Sample Preparation and Electron Cryomicroscopy

In order to obtain the most well-ordered complex possible, we wanted to use the finding that RNA structure and particularly pseudoknots appear to be more stable at lower pH (Li and Breaker, 1999; Nixon and Giedroc, 2000). In doing so, we used the fact that CrPV-IRES is not particularly species specific because it has been shown to function in the yeast *S. cerevisiae* (Thompson et al., 2001). This motivated us to use ribosomes from *Kluyveromyces lactis*, a yeast that grows at low pH (Rodicio and Heinisch, 2013) and shares a high degree of sequence similarity with *S. cerevisiae*, particularly of ribosomes and translation factors. Ribosomes from *K. lactis* are well behaved at pH 6, whereas at the same pH, ribosomes from *S. cerevisiae* showed aggregation. As expected, we found that CrPV-IRES forms a stable complex with ribosomes from *K. lactis.* As we show below, the complex also appears functional in interaction with elongation factors. The structure of the complex was determined by single-particle cryoelectron microscopy essentially as described previously for the yeast ribosome (Bai et al., 2013).

CrPV-IRES Is Bound to Both the Canonical and Rotated States of the Ribosome

An initial reconstruction yielded an overall resolution of 3.5 Å (Figures S1 (top) and S2 available online). However, the map showed that there was considerable variability in the conformation of the small ribosomal subunit (40S), the CrPV-IRES (located at the intersubunit space), and the L1-stalk of the large ribosomal subunit. Maximum-likelihood particle sorting (Scheres, 2012b) was applied recursively to the data set, and several homogeneous subpopulations were clearly identified. Two major subpopulations corresponded to a rotated state of the ribosomal subunits (20%) and a ribosome in a canonical state (12.6%), with resolutions of 3.7 and 3.8 Å, respectively (Figures S1 (middle) and S2). These two states are similar to the canonical and rotated states of the ribosome observed during translocation (e.g., Agirrezabala et al., 2008; Julián et al., 2008; Tourigny et al., 2013). In both states, the CrPV-IRES folds into a well-defined 3D structure in the intersubunit space of the ribosome, in agreement with previous studies (Schüler et al., 2006).

Because the overall refinement is dominated by the large subunit, we improved the quality of 40S and CrPV-IRES density by applying a soft mask to the volume comprising the 40S, the CrPV-IRES, and the L1-stalk in the reconstruction corresponding to the rotated state (Figure S1). Reclassification and refinement of this subpopulation in the presence of the mask allowed us to improve the definition of the density for the CrPV-IRES and its interaction with the 40S at the expense of a concomittant blurring of the 60S density (Figure S1, bottom). In combination with a similar masked map for the 60S subunit, the density was good enough (Figure 1B) to build and refine both subunits and the CrPV-IRES to obtain a complete atomic model for the CrPV-IRES in complex with the ribosome (Figures 1C and 1D). Model building was greatly facilitated by the crystal structure of the *S. cerevisiae* 80S ribosome (Ben-Shem et al., 2011), which has a small number of differences in sequence from the *K. lactis* ribosome used here. Many of these sequence differences were apparent in the density. Table S1 shows the refinement statistics, and Figure S3 shows the correlation of the final model with the map.

The rotated state of the ribosome differs from the canonical state by a counterclockwise rotation of the small subunit, including an additional movement of the head, as well as a movement of the L1 stalk (Figure 2A). Because the CrPV-IRES maintains interactions with each subunit in both states, the rotation of the subunits results in a somewhat different orientation and conformation for the CrPV-IRES in the two states (Figure 2B).

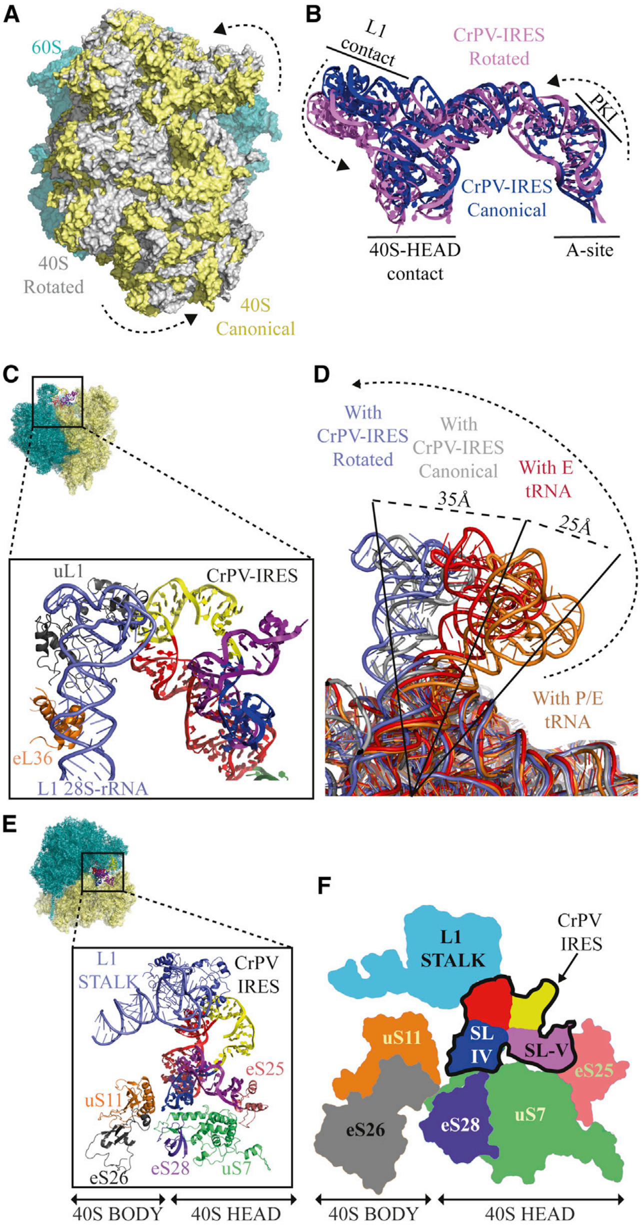

Figure 2. General Views of the CrPV-IRES/80S Complexes and Ratcheting Movements

(A) Superposition of the canonical (yellow) and rotated (gray) states of the 40S subunit showing a counter-clockwise rotation.

(B) Conformation of the CrPV-IRES in the rotated and canonical states of the ribosome in (A).

(C) Interaction of the CrPV-IRES with the L1 stalk of the 60S subunit.

(D) The conformation of the L1 stalk in different states of the ribosome. The stalk conformation with CrPV-IRES is from this work, whereas those with E-site tRNA and a hybrid P/E tRNA are from bacterial crystal structures (Gao et al., 2009; Tourigny et al., 2013).

(E and F) Ribbon diagram (left) and schematic (right) showing the simultaneous interaction of CrPV-IRES with the L1 stalk in the 60S subunit and elements of the 40S subunit.

Interaction with the L1 Stalk

The L1-stalk is a highly conserved feature of the large subunit and consists of a long stretch of 28S rRNA capped by ribosomal protein L1 (Figure 2C). This stalk is known to change its conformation in a manner that is coupled to tRNA movement during translocation (Figure 2D) (Fei et al., 2008; Gao et al., 2009; Tourigny et al., 2013). Although the structure of the stalk has been determined in various conformations in bacterial ribosomes, it is disordered in crystal structures of both the 80S ribosome (Ben-Shem et al., 2011) and 60S subunit (Klinge et al., 2011) because of its dynamic nature in the absence of interactions with tRNA. However, in the structures here, its orientation is fixed by its interactions with the L1.1 region of the CrPV-IRES, making it possible to completely build the eukaryotic L1 stalk (Figure 2C).

In normal translocation, the rotated state of the ribosome is accompanied by an inward movement of the L1 stalk, which thereby stabilizes a hybrid P/E tRNA state (Figure 2D) (Tourigny et al., 2013). In contrast, the interaction with CrPV-IRES in the rotated state stabilizes the stalk in an opposite outward conformation (Figures 2C and 2D). In this highly displaced position, the L1-stalk makes interactions with the C-terminal domain of protein eL36 in the main body of the large ribosomal subunit (Figure 2C). Even in the canonical state, the L1 stalk is displaced outward relative to the same state with normal tRNAs (Figure 2D).

Conformational Flexibility of the CrPV-IRES

The CrPV-IRES also interacts with the top of the 40S subunit through elements SL-IV and SL-V (Figure 1C). These interactions involve a cluster of proteins in the back of the 40S subunit, especially protein uS7, which bridges SL-IV and SL-V (Figures 2E and 2F) and are consistent with previous biochemical data (Jan and Sarnow, 2002; Nishiyama et al., 2007).

Because the L1 stalk and the head of the 40S both move relative to each other between the two states, the CrPV-IRES adopts a different conformation in the two states in order to maintain these interactions (Figure 2B). The conformation of the CrPV-IRES is thus coupled to the state of the ribosome, namely canonical or rotated, and to the conformation of the L1 stalk. This flexibility of the IRES is reminiscent of the flexibility of tRNAs as they spontaneously change conformation from canonical to hybrid states during translocation (Agirrezabala et al., 2008; Julián et al., 2008; Tourigny et al., 2013). This suggests that the CrPV-IRES-ribosome complex may be in an equilibrium between the canonical and rotated states, similar to tRNAs in the ribosome during translocation (Blanchard et al., 2004).

PKI Interactions with the Decoding Center of the 40S Subunit

The CrPV-IRES spans all three tRNA binding sites of the ribosome (Figures 3A and 3B). PKI is inserted in the decoding center of the 40S ribosomal A site (Figures 3B and 3C). It is somewhat tilted toward the P site relative to A site tRNA (Figure 3B), thus more closely resembling a tRNA in the A/P hybrid state characteristic of a translocation intermediate. The interaction of PKI in the decoding center of the 40S subunit is very similar to that reported for normal decoding of tRNA in the A site (Ogle et al., 2001) and involves an interaction of the conserved 18S rRNA bases A1756 and A1755 (1493/92 in *E. coli* numbering) and

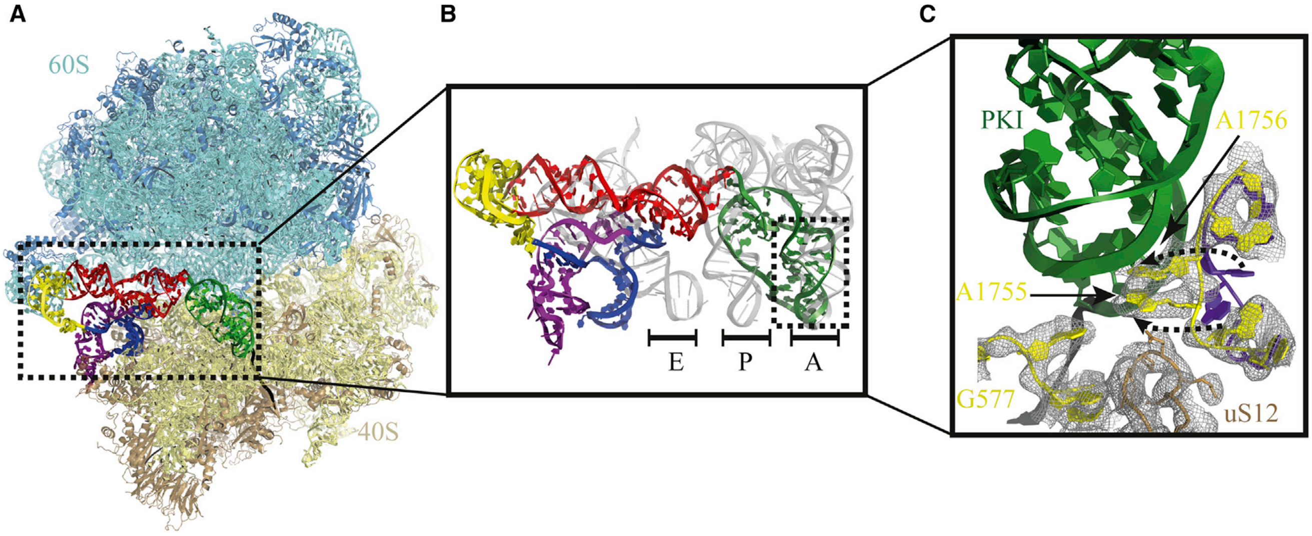

Figure 3. Interaction of the CrPV-IRES with the Decoding Center of the 40S Subunit

(A) Overview of the CrPV-IRES-ribosome.

(B) Superposition of the CrPV-IRES with A, P, and E site tRNAs (from 2J00, Selmer et al., 2006), showing that the green pseudoknot PKI of the IRES is in the decoding center normally occupied by an A site tRNA anticodon stem loop.

(C) Details of the interaction of PKI of the IRES showing that the three highly conserved 18S rRNA bases G577, A1755, and A1756 (corresponding to G530, A1492, and A1493 in *E. coli*) change conformation from relative to the structure of the empty yeast ribosome (purple; Ben-Shem et al., 2011). In doing so, they make the same interactions with the minor groove of PKI as during decoding of tRNA (Ogle et al., 2001).

G577 (530 in *E. coli*) with the minor groove of the elements that mimic the codon and anticodon in PKI. This shows that PKI mimics a decoding event in the A site.

The observation of PKI in the decoding center in both states of the ribosome has implications for initiation of translation by CrPV-IRES. Because the A site is already occupied by PKI and the first codon of the mRNA is not in the A site but just downstream of it (Figures 1C and 1D), the ribosome cannot start translation on the first codon by binding a ternary complex of the cognate aminoacyl tRNA and eEF1A in the A site. Rather, translocation would be required to move PKI into the P site, which would result in the first codon being placed in the A site and able to bind a cognate ternary complex. To test this hypothesis, we tested the binding of elongation factors to the CrPV-IRES before and after translocation by eEF2.

The CrPV-IRES Ribosome Complex Requires a Translocation Event before It Can Accept an Aminoacyl tRNA

Elongation in eukaryotes requires the two GTPase factors eEF1A and eEF2, which are the eukaryotic homologs of EF-Tu and EF-G, respectively. Of these, eEF1A delivers incoming aminoacyl tRNAs as part of a ternary complex with GTP, and eEF2 in the GTP form binds a rotated form of the ribosome and facilitates translocation (Figure 4A). If the CrPV-IRES mimics a pretranslocation state, it should not be able to bind ternary complex initially but should be able to do so after translocation by eEF2 (Figure 4B).

The binding of tRNA-eEF1A or eEF2 to the CrPV-IRES-ribosome complex was tested by copelleting experiments. In these experiments, the first codon of the coding sequence of the CrPV-IRES mRNA was mutated to phenylanine to make it cognate to the yeast Phe-tRNA^{Phe} used in the ternary complex with eEF1A. The GTP analog GDPCP was used to trap the factors bound to the ribosome. The factors will only copellet with the ribosome if they can bind stably to it. As would be expected from the structure, we could see good binding of eEF2 (Figure 4C, left gel, lane 4) but no binding of the ternary complex of eEF1A to the CrPV-IRES-ribosome complex (Figure 4C, left gel, lane 5). This is consistent with the notion that the A site is blocked by PKI in the absence of translocation but that eEF2 can bind and stabilize the pretranslocational state seen in the structure.

To see whether translocation of the IRES would allow binding of the ternary complex to the ribosome, the CrPV-IRES-ribosome complex was first incubated with eEF2 in the presence of GTP to facilitate translocation and pelleted through a sucrose cushion as above to remove GTP and excess eEF2. Subsequently, the ternary complex of eEF1A and aminoacyl tRNA in the presence of GDPCP was added and copelleted as above. In this case, eEF1A copellets with the CrPV-IRES complex (Figure 4C, right gel). Although there is some residual eEF2 from a small fraction of ribosomes that had not undergone translocation, the presence of copelleting eEF1A is in contrast to the complete absence of ternary complex binding prior to translocation. Figure 4D shows a western blot of a gel run exactly as in 4C but probed with an antibody to the calmodulin binding protein tag on the factors, thus confirming their identity.

Together with the structure, these experiments show that the CrPV-IRES is initially a substrate for eEF2 binding, but not ternary complex binding, and it is only after translocation with eEF2 that it is able to bind ternary complex.

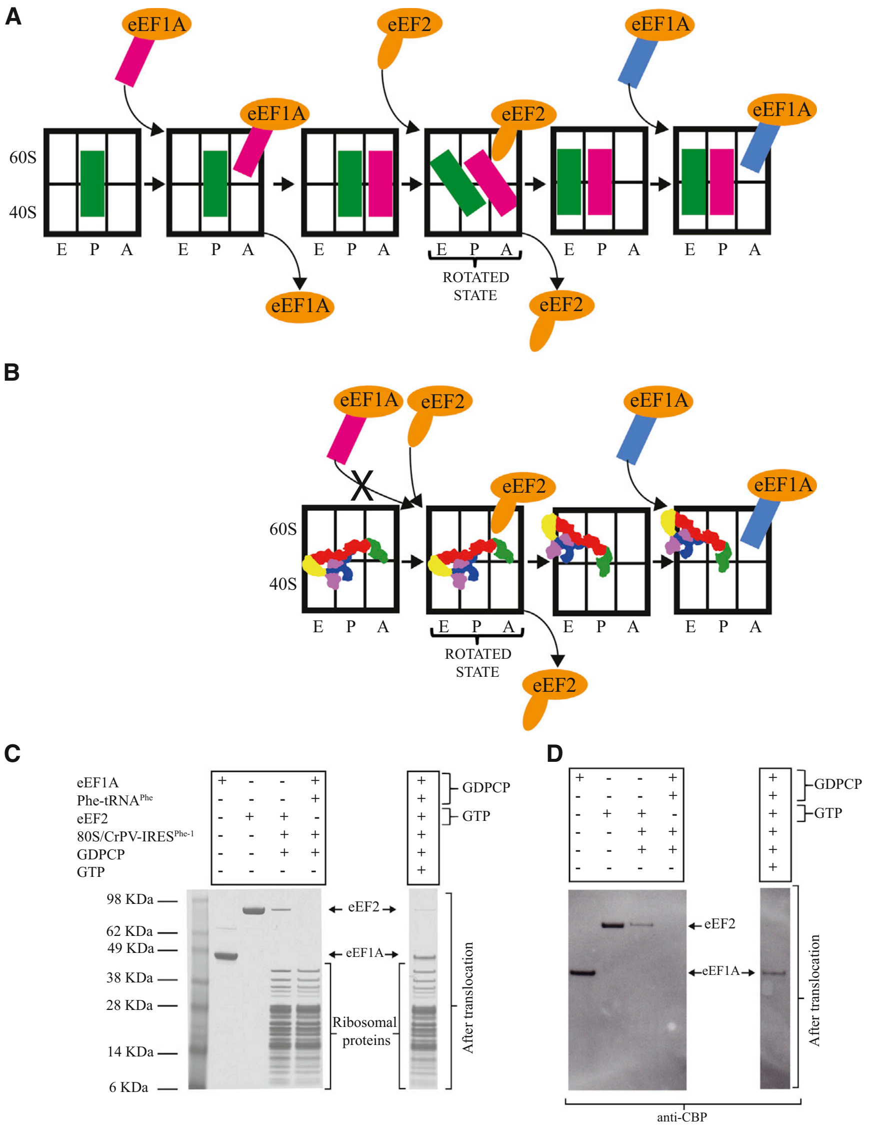

Figure 4. The CrPV-IRES Mimics a Pretranslocational State

(A) Scheme showing the action of the ternary complex of eEF1A, tRNA, and eEF2 during the normal elongation cycle. After initiation, a cognate ternary complex binds to the empty A site and delivers an aminoacyl tRNA. After dissociation of eEF1A, this pretranslocational complex is a substrate for eEF2 binding, which stabilizes a hybrid state and catalyzes translocation of the tRNAs. Following translocation, a second round of binding of ternary complex can take place.

(B) Because the ribosome-CrPV-IRES complex has PKI in the A site, it should not be able to bind eEF1A and tRNA. However, it should be able to bind eEF2, and, following translocation and dissociation of eEF2, eEF1A and tRNA should be able to bind to the now vacated A site.

(C) The binding of factors eEF1A (with cognate Phe-tRNA^{Phe}) or eEF2 to the ribosome-CrPV-IRES complex was tested both before and after translocation by copelleting experiments. The last two lanes on the gel on the left show that eEF2 with the GTP analog GDPCP can bind to the ribosome-CrPV-IRES complex, but the ternary complex of eEF1A and tRNA does not bind. However, the gel on the right shows that, after translocation by eEF2 in the presence of GTP, the ternary complex of eEF1A, tRNA, and GDPCP can bind. The presence of some residual eEF2 in this lane is due to a small fraction of untranslocated ribosomes.

(D) Western blot of a gel run exactly as in (C) but probed with an antibody to the calmodulin-binding protein tag on the factors.

DISCUSSION

A key step in normal initiation involves placing initiator tRNA and the start codon in the P site. Because CrPV-IRES can initiate translation in the absence of any initiation factors and contains a pseudoknot PKI just upstream of the first codon that is a structural mimic of tRNA/mRNA interaction (Costantino et al., 2008), it was logical to conclude that the binding of CrPV-IRES to the ribosome places PKI in the P site, thereby mimicking initiator tRNA. Indeed, early biochemical studies suggested that this was the case (Wilson et al., 2000; Pestova and Hellen, 2003). This scheme would also place the first codon in the A site, so the ribosome would be ready to accept a cognate tRNA.

In this work, we have determined a high-resolution structure and refined an atomic model for the CrPV-IRES in both the canonical and rotated states. In both states, PKI is clearly located in the decoding center. Moreover, because the elements that correspond to the anticodon and codon in PKI are correctly base paired, they are recognized by the decoding center in the same way as a cognate tRNA/mRNA, including an interaction of the conserved bases of the decoding center with the minor groove of PKI (Figure 3C).

The presence of both canonical and rotated states suggests that CrPV-IRES is able to exist in an equilibrium between the canonical and the rotated states of the ribosome, like tRNAs after peptidyl transfer (Blanchard et al., 2004). This state is a substrate for the binding of eEF2, which, like its bacterial homolog EF-G, presumably also stabilizes the hybrid state prior to translocation (Spiegel et al., 2007). It is only after translocation of the IRES by eEF2 that PKI can move into the P site, thus bringing the first codon of the mRNA into the A site for recognition of a cognate tRNA brought as a ternary complex by eEF1A and GTP.

Prior biochemical experiments measured translocation using toe-printing experiments (e.g., Jan et al., 2003; Pestova and Hellen, 2003). We note that, in most of these experiments, eEF2 was generally present when eEF1A and tRNA were included, so it would not have been possible to know whether eEF2 acted both before and after binding of eEF1A/tRNA. Indeed, one puzzle in these experiments was that addition of tRNA, eEF1A and eEF2 caused a shift of six rather than three nucleotides, which would

indicate two translocation events rather than one. Moreover, whereas toe-printing is very useful to measure relative movement, the absolute determination of the mRNA nucleotides in the P or A site requires calibration in each case. We should point out, however, that no shift in the toe-print was observed in some experiment in which only eEF2 was added without eEF1A or tRNA (Pestova and Hellen, 2003; Jan et al., 2003). On the face of it, this result would be incompatible with our conclusions. However, it is possible that the CrPV-IRES is capable of spontaneous back translocation and prefers the pretranslocated state unless eEF1A and tRNA bind in a short time interval after the first translocation event by eEF2. Further experiments are needed to clarify this discrepancy.

The previous structure of the CrPV-IRES places the molecule in roughly the same position as in our structure (Schüler et al., 2006). However, besides being at considerably lower resolution, "Domain 3," which includes PKI, had weaker density than the rest of the molecule. Unlike their previous 40S-CrPV-IRES complex (Spahn et al., 2004), in which Domain 3 was placed entirely in the A site, they suggest that, in the 80S ribosome, it is located between the A and P sites with only the apical stem overlapping with the anticodon stem loop of A site tRNA. They also suggest that the weaker density may be a consequence of flexibility, which would allow it to move out of the A site. Because PKI is well ordered in both our structures, it is possible that their structure was a superposition of the two states we see because modern classification methods were not generally in use then. This superposition may have resulted in an ambiguity in their density for PKI. An isolated domain of the IRES, including PKI, was placed in the P site of a bacterial ribosome structure (Zhu et al., 2011). However, tRNA binding to an empty bacterial ribosome prefers the P site, so this structure does not represent the initial binding of a full-length IRES to a eukaryotic ribosome. Indeed, in their modeling of the CrPV-IRES into the cryo-EM density of Schüler et al. (2006), the authors place PKI in the A site and recognize that it needs to move subsequently into the P site. It could also be argued that our structure may not represent the situation in vivo because we have used a heterologous system with ribosomes from *K. lactis*. We consider this unlikely because CrPV-IRES was shown to function in vivo in yeast (Thompson et al., 2001). Moreover, the intersubunit region of the eukaryotic ribosome consists almost entirely of RNA, is highly conserved, and has to accommodate a variety of tRNAs in normal translation, so we think it is unlikely that CrPV-IRES binds differently to this region of the ribosome in different species.

Independent support for our work comes from experiments on the intergenic region IRES from *Plautia stali* intestine virus (PSIV-IRES), which also contains a pseudoknot just upstream of the first codon of the translated region. This IRES requires a translocation event by eEF2 before it can bind the first cognate tRNA delivered by eEF1A (Yamamoto et al., 2007), just as we propose for CrPV-IRES here, and suggests that the two IRESs work in the same way.

The elongation cycle is much more highly conserved than initiation, which diverges considerably among domains of life. By mimicking a key step in the elongation cycle, these and similar IRESs are taking advantage of the conserved intersubunit space and intrinsic dynamic properties of the ribosome. This also allows them to completely circumvent the regulation of initiation by the host cell. Moreover, because it uses only a folded RNA and the ribosome itself, such a mechanism could well have existed before the evolution of a complex initiation pathway. In any case, our structure shows that CrPV-IRES mimics a pretranslocational rather than an initiation state of the ribosome.

EXPERIMENTAL PROCEDURES

Preparation of Ribosomes

Ribosomes were purified from the *K. lactis* strain GG799. Cells were harvested in mid-log phase (OD_{600} of 2–4) and resuspended in 20 mM MES-KOH (pH 6), 150 mM KCl, 150 mM K-acetate, 10 mM Mg^{+2} acetate, 1 mg/ml heparin, 0.1 mM PMSF, 0.1 mM benzamidine, and 2 mM DTT. Cell pellets frozen in liquid nitrogen were mechanically disrupted by a blender. The lysate was thawed at 4°C and clarified by centrifugation for 20 min at 14,500 g. Ribosomes in the supernatant were pelleted through a 1 M sucrose cushion in the same buffer for 4 hr at 45,000 rpm in a Ti45 rotor (Beckman Coulter). The pellets were resuspended in 20 mM MES-KOH (pH 6), 50 mM KCl, 5 mM Mg^{+2} acetate, 0.1 mM PMSF, 0.1 mM benzamidine, and 2 mM DTT and incubated for 15 min with 1 mM puromycin on ice. The sample was loaded on a 10%–40% sucrose gradient in the same resuspension buffer and centrifuged for 16 hr at 28,000 rpm in a Ti25 zonal rotor (Beckman Coulter). For subunit purification, 80S ribosomes were exchanged into dissociation buffer (20 mM MES-KOH [pH 6], 600 mM KCl, 8 mM Mg^{+2} -acetate, 1 mg/ml heparin, 0.1 mM PMSF, 0.1 mM benzamidine, and 2 mM DTT) before loading onto a sucrose gradient in the same buffer and centrifuged for 19 hr at 28,500 rpm in the Ti25 rotor. The 60S and 40S subunits were exchanged separately into reassociation buffer (10 mM MES-KOH [pH 6], 10 mM NH_4 acetate, 40 mM K acetate, 8 mM Mg^{+2} acetate, and 2 mM DTT), concentrated to 6 μM, and stored at −80°C after being flash frozen in liquid nitrogen.

Preparation of CrPV IRES

The genes for wild-type CrPV-IRES and a mutant CrPV-IRES^{Phe-1}, in which the first codon of the message was replaced by one for phenylananine, were chemically synthetized and cloned in the pUC19 vector flanked at the 5′ by a T7 promoter sequence and an EcoRI cleavage site at the 3′. Standard in vitro transcription protocols (Price et al., 1995) were used for the production of the IRES RNA in each case.

A 3× molar excess of CrPV-IRES (240 nM) was mixed with the 40S subunit (80 nM) in reassociation buffer (10 mM MES-KOH [pH 6], 40 mM K-acetate, 10 mM NH4-acetate, 8 mM Mg+2-acetate, and 2 mM DTT) in a total volume of 25 μl and incubated for 2 min at 30°C before the addition of the 60S to a final concentration of 80 nM. A final incubation of 5 min at 30°C was performed before the sample was cooled to 4°C and used immediately to make cryo-EM grids.

Purification of Elongation Factors and tRNA

The genes for elongation factors eEF1A and eEF2 from *K. lactis* were cloned and overexpressed in *S. cerevisiae* strain YAS-2488 as described previously (Galej et al., 2013). The crude extract after cell disruption was adjusted to pH 8 with Tris base and centrifuged at 48,000 *g* at 4°C for 30 min. The supernatant was incubated with calmodulin-sepharose (recombinant calmodulin coupled to cyanogen bromide-activated sepharose [GE]) overnight at 4°C. The resin was washed with CAL500W buffer (20 mM Tris-HCl [pH 8], 500 mM NaCl, 2 mM CaCl2, 1 mM Mg-acetate, 1 mM imidazole, and 10 mM 2-mercaptoethanol) and eluted with CAL500E buffer (20 mM Tris-HCL [pH8.0], 500 mM NaCl, 2 mM EGTA, 1 mM Mg-acetate, 1 mM imidazole, and 10 mM 2-mercaptoethanol). After dialysis against Low-Salt-dialysis buffer (20 mM Tris-HCl [pHc7.2], 50 mM KCl, 10 mM NH_4Cl, 1 mM $MgCl_2$, and 2 mM DTT) at 4°C, the samples were further purified by ionic exchange (SP-HP-sepharose column for eEF1A and Q-HP-sepharose column for eEF2) and gel filtration (sephacryl-S100 for eEF1A and sephacryl-S200 for eEF2). The running buffer for the final gel filtration step was 20 mM HEPES-KOH [pH 7.45], 200 mM K-acetate, 10 mM NH_4Cl, 1 mM $MgCl_2$, and 2 mM DTT).

eEF1A and eEF2 eluted from the gel filtration step were concentrated to 9.6 and 3.7 μM, respectively, snap frozen in liquid nitrogen, and stored at −80°C.

Phe-tRNA^{Phe} was generated by acylation of commercially available yeast tRNA^{Phe} (Sigma) as previously described (Nissen et al., 1995).

Factor Binding Studies

The ribosome-IRES complex formed with 1 μM ribosomes and 3 μM of IRES in reassociation buffer (10 mM MES-KOH [pH 6], 40 mM K acetate, 10 mM NH4 acetate, 8 mM Mg+2 acetate, and 2 mM DTT) was incubated at room temperature for 5 min with either 3 μM eEF1A and 12 μM of Phe-tRNA^{Phe} or 3 μM of eEF2, both in the presence of 100 μM of GDPCP. For copelleting, 50 μl of the mixture was layered on top of a 1 ml cushion of 1.1 M sucrose in reassociation buffer and centrifuged for 14 hr at 45,000 rpm and 4°C in a TLA 100.3 rotor (Beckman Coulter). The pellets were resuspended in 50 μl reassociation buffer.

For binding of ternary complex after translocation, the ribosome-IRES mixture was first incubated with eEF2 as above but in the presence of GTP instead of GDPCP. The ribosome complex was then pelleted through a sucrose cushion as above to remove GTP and excess eEF2 and resuspended in the same buffer. Subsequently, ternary complex of eEF1A, Phe-tRNA^{Phe}, and GDPCP was added as above and incubated for a further 5 min, and the final mixture was analyzed by copelleting as above.

Resuspended pellets from each copelleting experiment were analyzed by electrophoresis using 4%–12% gradient SDS-polyacrylamide gel. Gels were either stained in Coomassie blue or probed with antibodies to calmodulin binding protein (CBP) tag used in the cloning of the factors. For the western blot, anti-CBP-Rabbit antibodies (Genscript) and secondary goat-anti-Rabbit coupled to horseradish peroxidase antibody (abCam) were both used at 1:10,000 dilution. For detection, the ECL western blot substrate (Pierce) was used following the manufacturer's instructions.

Electron Microscopy

Aliquots of 3 μl of the CrPV-IRES/80S initiation complex at a concentration of~80 nM were incubated for 30 s on glow-discharged holey carbon grids (Quantifoil R2/2), on which a homemade continuous carbon film (estimated to be ~30 Å thick) had previously been deposited. Grids were blotted for 2.5 s and flash cooled in liquid ethane using an FEI Vitrobot. Grids were transferred to an FEI Titan Krios microscope that was operated at 300 kV. Defocus values in the final data set ranged from 1.6–3.6 μm. Images were recorded manually and in automatic mode on a back-thinned FEI Falcon II detector at a calibrated magnification of 104,478 (yielding a pixel size of 1.34 Å) as described previously (Bai et al., 2013). All electron micrographs that showed signs of significant astigmatism or drift were discarded.

Analysis and Structure Determination

All reconstructions described were calculated using semiautomated image processing as outlined below. We used the swarm tool in the e2boxer.py program of EMAN2 (Tang et al., 2007) for semiautomated particle picking. For our final data set, we selected 193,297 particles from 1,012 micrographs. Contrast transfer function parameters were estimated using CTFFIND (Mindell and Grigorieff, 2003). All 2D and 3D refinements were performed using RELION (Scheres, 2012a).

We used a reference-free 2D class averaging to discard 60S subunits and defective particles, resulting in 144,619 particles of the final data set for subsequent 3D classification and refinement. Refinement of all particles against the yeast 80S crystal structure low-pass filtered to 40 Å resolution yielded a preliminary, consensus reconstruction with local fuzzy density for the 40S subunit, the L1 stalk, and the CrPV-IRES. Subsequently, we employed a cascaded 3D classification scheme (Figure S1) to identify three major classes of different ratcheting states that were later reclassified and rerefined independently. Only two of the three classes were homogeneous enough to yield atomic resolution reconstructions, and these were analyzed further. To further increase the resolution of both classes, we performed statistical movie processing as described previously (Bai et al., 2013). In this procedure, we used running averages of five movie frames; an SD of 1° for the priors on the Euler angles; and an SD of 1 pixel for the translations. Reported resolutions (Figure S2) are based on the gold-standard FSC = 0.143 criterion, which is a realistic estimate of the resolution in the absence of overfitting (Scheres and Chen, 2012). Local resolution was estimated using the program ResMap (Kucukelbir et al., 2013). Prior to visualization, all density maps were corrected for the modulation transfer function (MTF) of the detector and then sharpened by applying a negative B factor (−217 Å^2) that was estimated using automated procedures (Rosenthal and Henderson, 2003).

Rigid-body fitting of the coordinates of the *S. cerevisiae* 80S ribosome (PDB ID 3U5B and 3U5D [Ben-Shem et al., 2011]) using Chimera (Goddard et al., 2007) was used initially, placing independently the 60S, the 40S head, and the 40S-body. Further improvement of the fitting of individual proteins and segments of rRNA was done with Coot (Emsley et al., 2010). The experimental density showed excellent agreement with the fitted model. In much of the interior, it was possible to identify individual side chains. For building the CrPV-IRES, the starting model used was from a previous lower-resolution EM structure (PDB ID 2NOQ); (Schüler et al., 2006), except that, for PKI, the crystal structure of this segment in isolation was used (PDB ID 3B31); (Costantino et al., 2008). Model building and refinement were carried out using Coot (Emsley et al., 2010) and Refmac (Murshudov et al., 1997) as recently described (Amunts et al., 2014). For refinement of the atomic model, masked areas of the canonical map corresponding to the 60S and the 40S/IRES were used independently. After customizing the refinement parameter for these sections, the rotated maps were interpreted using these refined models fitted as rigid bodies.

To prevent overfitting, the weights for refinements were carefully adjusted by cross-validation (Figure S3). The optimal weight in REFMAC between the geometric restraints and the fit to experimental density was determined empirically to minimize overfitting. This procedure involved the use of both "half maps" that were calculated from the same halves of the particles as used for the gold-standard FSC calculations. To remove model bias from the model that was built in a reconstruction from all particles, the atoms were randomly displaced by up to a maximum of 0.5 Å before a full refinement using secondary structure, base pair, and planarity restraints was performed against one of the two half maps. For each refinement, in addition to calculating the FSC between the refined model and the map it was refined against (the FSC_{work}, red curves in Figure S3), we also calculated a cross-validated FSC between the refined model and the other half map (the FSC_{test} curve green in Figure S3). Putting too much weight on the fit to the density resulted in low R factors and high FSCs but relatively poor geometry. In such refinements, large differences between FSC_{work} and FSC_{test} and the sudden drop in FSC_{work} at the highest resolution that was used in refinement are clear indications of substantial overfitting. At the other extreme of putting very little weight on the fit to the density, models with excellent geometry but higher R factors and lower FSCs were obtained, and practically no differences between FSC_{work} and FSC_{test} were observed. Thereby, the half-map refinements allowed us to determine an optimal weight for each subunit, where good geometry and small differences between FSC_{work} and FSC_{test} were balanced against good fits to the density. The optimal weights were then used for the final refinement of each subunit against a reconstruction from all particles.

ACCESSION NUMBERS

The cryo-EM density maps have been deposited in the EMDB with accession numbers EMD-2603 and EMD-2604 (for the ratcheted unmasked and masked class maps, respectively) and EMD-2599 (for the canonical class). Fitted atomic coordinates have been deposited in the PDB with entry codes 4cuv, 4cuw for the canonical, and 4cux and 4cuy for the ratcheted states.

SUPPLEMENTAL INFORMATION

Supplemental Information includes three figures and one table and can be found with this article online at http://dx.doi.org/10.1016/j.cell.2014.04.015.

ACKNOWLEDGMENTS

We are grateful to Shaoxia Chen for technical support with cryo-EM, Greg McMullan for help in movie data acquisition, Toby Darling and Jake Grimmett for help with computing, David Tourigny and Alan Brown for discussions,

Ann C. Kelley for advice and help in RNA production, and Wojciech Galej and Andrew Newman for advice in yeast protein expression. X.-C.B. is supported by an EU FP7 Marie Curie postdoctoral fellowship. This work was funded by grants from the UK Medical Research Council (MC_U105184332 to V.R. and MC_UP_A025_1013 to SHWS) and a Wellcome Trust Senior Investigator award (WT096570), the Agouron Institute, and the Jeantet Foundation (V.R.).

Received: February 17, 2014
Revised: March 14, 2014
Accepted: April 11, 2014
Published: May 1, 2014

REFERENCES

Agirrezabala, X., Lei, J., Brunelle, J.L., Ortiz-Meoz, R.F., Green, R., and Frank, J. (2008). Visualization of the hybrid state of tRNA binding promoted by spontaneous ratcheting of the ribosome. Mol. Cell *32*, 190–197.

Aitken, C.E., and Lorsch, J.R. (2012). A mechanistic overview of translation initiation in eukaryotes. Nat. Struct. Mol. Biol. *19*, 568–576.

Amunts, A., Brown, A., Bai, X.-C., Llácer, J.L., Hussain, T., Emsley, P., Long, F., Murshudov, G., Scheres, S.H.W., and Ramakrishnan, V. (2014). Structure of the yeast mitochondrial large ribosomal subunit. Science *343*, 1485–1489.

Bai, X.C., Fernandez, I.S., McMullan, G., and Scheres, S.H. (2013). Ribosome structures to near-atomic resolution from thirty thousand cryo-EM particles. Elife *2*, e00461.

Ben-Shem, A., Garreau de Loubresse, N., Melnikov, S., Jenner, L., Yusupova, G., and Yusupov, M. (2011). The structure of the eukaryotic ribosome at 3.0 Å resolution. Science *334*, 1524–1529.

Blanchard, S.C., Kim, H.D., Gonzalez, R.L., Jr., Puglisi, J.D., and Chu, S. (2004). tRNA dynamics on the ribosome during translation. Proc. Natl. Acad. Sci. USA *101*, 12893–12898.

Costantino, D.A., Pfingsten, J.S., Rambo, R.P., and Kieft, J.S. (2008). tRNA-mRNA mimicry drives translation initiation from a viral IRES. Nat. Struct. Mol. Biol. *15*, 57–64.

Emsley, P., Lohkamp, B., Scott, W.G., and Cowtan, K. (2010). Features and development of Coot. Acta Crystallogr. D Biol. Crystallogr. *66*, 486–501.

Fei, J., Kosuri, P., MacDougall, D.D., and Gonzalez, R.L.J., Jr. (2008). Coupling of ribosomal L1 stalk and tRNA dynamics during translation elongation. Mol. Cell *30*, 348–359.

Filbin, M.E., and Kieft, J.S. (2009). Toward a structural understanding of IRES RNA function. Curr. Opin. Struct. Biol. *19*, 267–276.

Galej, W.P., Oubridge, C., Newman, A.J., and Nagai, K. (2013). Crystal structure of Prp8 reveals active site cavity of the spliceosome. Nature *493*, 638–643.

Gao, Y.G., Selmer, M., Dunham, C.M., Weixlbaumer, A., Kelley, A.C., and Ramakrishnan, V. (2009). The structure of the ribosome with elongation factor G trapped in the posttranslocational state. Science *326*, 694–699.

Goddard, T.D., Huang, C.C., and Ferrin, T.E. (2007). Visualizing density maps with UCSF Chimera. J. Struct. Biol. *157*, 281–287.

Jackson, R.J., Hellen, C.U., and Pestova, T.V. (2010). The mechanism of eukaryotic translation initiation and principles of its regulation. Nat. Rev. Mol. Cell Biol. *11*, 113–127.

Jan, E., and Sarnow, P. (2002). Factorless ribosome assembly on the internal ribosome entry site of cricket paralysis virus. J. Mol. Biol. *324*, 889–902.

Jan, E., Kinzy, T.G., and Sarnow, P. (2003). Divergent tRNA-like element supports initiation, elongation, and termination of protein biosynthesis. Proc. Natl. Acad. Sci. USA *100*, 15410–15415.

Julián, P., Konevega, A.L., Scheres, S.H., Lázaro, M., Gil, D., Wintermeyer, W., Rodnina, M.V., and Valle, M. (2008). Structure of ratcheted ribosomes with tRNAs in hybrid states. Proc. Natl. Acad. Sci. USA *105*, 16924–16927.

Kanamori, Y., and Nakashima, N. (2001). A tertiary structure model of the internal ribosome entry site (IRES) for methionine-independent initiation of translation. RNA *7*, 266–274.

Klinge, S., Voigts-Hoffmann, F., Leibundgut, M., Arpagaus, S., and Ban, N. (2011). Crystal structure of the eukaryotic 60S ribosomal subunit in complex with initiation factor 6. Science *334*, 941–948.

Kucukelbir, A., Sigworth, F.J., and Tagare, H.D. (2013). Quantifying the local resolution of cryo-EM density maps. Nat. Methods *11*, 63–65.

Li, Y., and Breaker, R.R. (1999). Kinetics of RNA degradation by specific base catalysis of transesterification involving the 2′-hydroxyl group. J. Am. Chem. Soc. *121*, 5364–5372.

Li, X., Mooney, P., Zheng, S., Booth, C.R., Braunfeld, M.B., Gubbens, S., Agard, D.A., and Cheng, Y. (2013). Electron counting and beam-induced motion correction enable near-atomic-resolution single-particle cryo-EM. Nat. Methods *10*, 584–590.

Marintchev, A., and Wagner, G. (2004). Translation initiation: structures, mechanisms and evolution. Q. Rev. Biophys. *37*, 197–284.

Mindell, J.A., and Grigorieff, N. (2003). Accurate determination of local defocus and specimen tilt in electron microscopy. J. Struct. Biol. *142*, 334–347.

Murshudov, G.N., Vagin, A.A., and Dodson, E.J. (1997). Refinement of macromolecular structures by the maximum-likelihood method. Acta Crystallogr. D Biol. Crystallogr. *53*, 240–255.

Nishiyama, T., Yamamoto, H., Uchiumi, T., and Nakashima, N. (2007). Eukaryotic ribosomal protein RPS25 interacts with the conserved loop region in a dicistroviral intergenic internal ribosome entry site. Nucleic Acids Res. *35*, 1514–1521.

Nissen, P., Kjeldgaard, M., Thirup, S., Polekhina, G., Reshetnikova, L., Clark, B.F., and Nyborg, J. (1995). Crystal structure of the ternary complex of Phe-tRNAPhe, EF-Tu, and a GTP analog. Science *270*, 1464–1472.

Nixon, P.L., and Giedroc, D.P. (2000). Energetics of a strongly pH dependent RNA tertiary structure in a frameshifting pseudoknot. J. Mol. Biol. *296*, 659–671.

Ogle, J.M., Brodersen, D.E., Clemons, W.M., Jr., Tarry, M.J., Carter, A.P., and Ramakrishnan, V. (2001). Recognition of cognate transfer RNA by the 30S ribosomal subunit. Science *292*, 897–902.

Pestova, T.V., and Hellen, C.U. (2003). Translation elongation after assembly of ribosomes on the Cricket paralysis virus internal ribosomal entry site without initiation factors or initiator tRNA. Genes Dev. *17*, 181–186.

Pfingsten, J.S., Costantino, D.A., and Kieft, J.S. (2006). Structural basis for ribosome recruitment and manipulation by a viral IRES RNA. Science *314*, 1450–1454.

Price, S.R., Ito, N., Oubridge, C., Avis, J.M., and Nagai, K. (1995). Crystallization of RNA-protein complexes. I. Methods for the large-scale preparation of RNA suitable for crystallographic studies. J. Mol. Biol. *249*, 398–408.

Rodicio, R., and Heinisch, J.J. (2013). Yeast on the milky way: genetics, physiology and biotechnology of Kluyveromyces lactis. Yeast *30*, 165–177.

Rosenthal, P.B., and Henderson, R. (2003). Optimal determination of particle orientation, absolute hand, and contrast loss in single-particle electron cryomicroscopy. J. Mol. Biol. *333*, 721–745.

Scheres, S.H. (2012a). RELION: implementation of a Bayesian approach to cryo-EM structure determination. J. Struct. Biol. *180*, 519–530.

Scheres, S.H. (2012b). A Bayesian view on cryo-EM structure determination. J. Mol. Biol. *415*, 406–418.

Scheres, S.H., and Chen, S. (2012). Prevention of overfitting in cryo-EM structure determination. Nat. Methods *9*, 853–854.

Schüler, M., Connell, S.R., Lescoute, A., Giesebrecht, J., Dabrowski, M., Schroeer, B., Mielke, T., Penczek, P.A., Westhof, E., and Spahn, C.M. (2006). Structure of the ribosome-bound cricket paralysis virus IRES RNA. Nat. Struct. Mol. Biol. *13*, 1092–1096.

Selmer, M., Dunham, C.M., Murphy, F.V., 4th, Weixlbaumer, A., Petry, S., Kelley, A.C., Weir, J.R., and Ramakrishnan, V. (2006). Structure of the 70S ribosome complexed with mRNA and tRNA. Science *313*, 1935–1942.

Spahn, C.M., Jan, E., Mulder, A., Grassucci, R.A., Sarnow, P., and Frank, J. (2004). Cryo-EM visualization of a viral internal ribosome entry site bound to

human ribosomes: the IRES functions as an RNA-based translation factor. Cell *118*, 465–475.

Spiegel, P.C., Ermolenko, D.N., and Noller, H.F. (2007). Elongation factor G stabilizes the hybrid-state conformation of the 70S ribosome. RNA *13*, 1473–1482.

Tang, G., Peng, L., Baldwin, P.R., Mann, D.S., Jiang, W., Rees, I., and Ludtke, S.J. (2007). EMAN2: an extensible image processing suite for electron microscopy. J. Struct. Biol. *157*, 38–46.

Thompson, S.R., Gulyas, K.D., and Sarnow, P. (2001). Internal initiation in Saccharomyces cerevisiae mediated by an initiator tRNA/eIF2-independent internal ribosome entry site element. Proc. Natl. Acad. Sci. USA *98*, 12972–12977.

Tourigny, D.S., Fernández, I.S., Kelley, A.C., and Ramakrishnan, V. (2013). Elongation factor G bound to the ribosome in an intermediate state of translocation. Science *340*, 1235490.

Wilson, J.E., Pestova, T.V., Hellen, C.U., and Sarnow, P. (2000). Initiation of protein synthesis from the A site of the ribosome. Cell *102*, 511–520.

Yamamoto, H., Nakashima, N., Ikeda, Y., and Uchiumi, T. (2007). Binding mode of the first aminoacyl-tRNA in translation initiation mediated by Plautia stali intestine virus internal ribosome entry site. J. Biol. Chem. *282*, 7770–7776.

Zhu, J., Korostelev, A., Costantino, D.A., Donohue, J.P., Noller, H.F., and Kieft, J.S. (2011). Crystal structures of complexes containing domains from two viral internal ribosome entry site (IRES) RNAs bound to the 70S ribosome. Proc. Natl. Acad. Sci. USA *108*, 1839–1844.

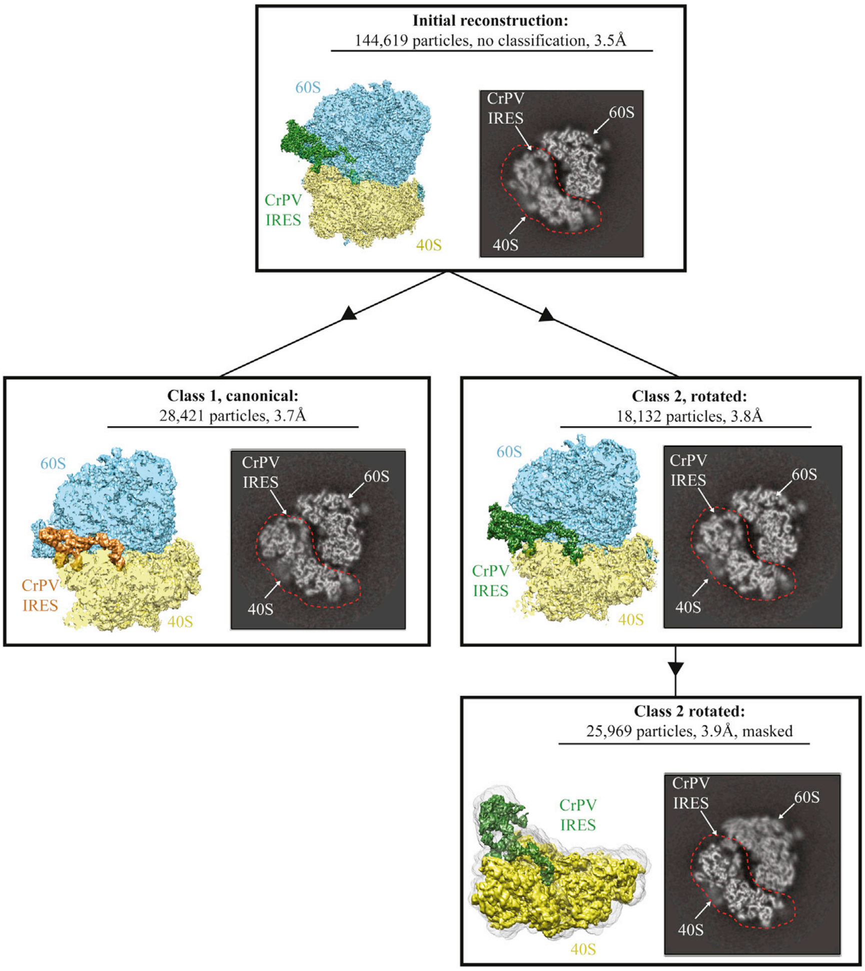

Figure S1. Overview of 3D Classification, Related to Figure 1

3D classification scheme used to obtain the complex of the CrPV-IRES with the ribosome in the canonical and rotated states. Although the initial reconstruction had a relatively high overall resolution, the 40S and IRES components were fuzzy as shown in a slice through the cryo-EM map shown on the right. There were two major classes present in the structure, corresponding to canonical and rotated states, which yielded improved density for the 40S and IRES (at the expense of some loss of overall resolution). Further improvement was obtained by a refinement in which a soft mask around the 40S and IRES was used for alignment.

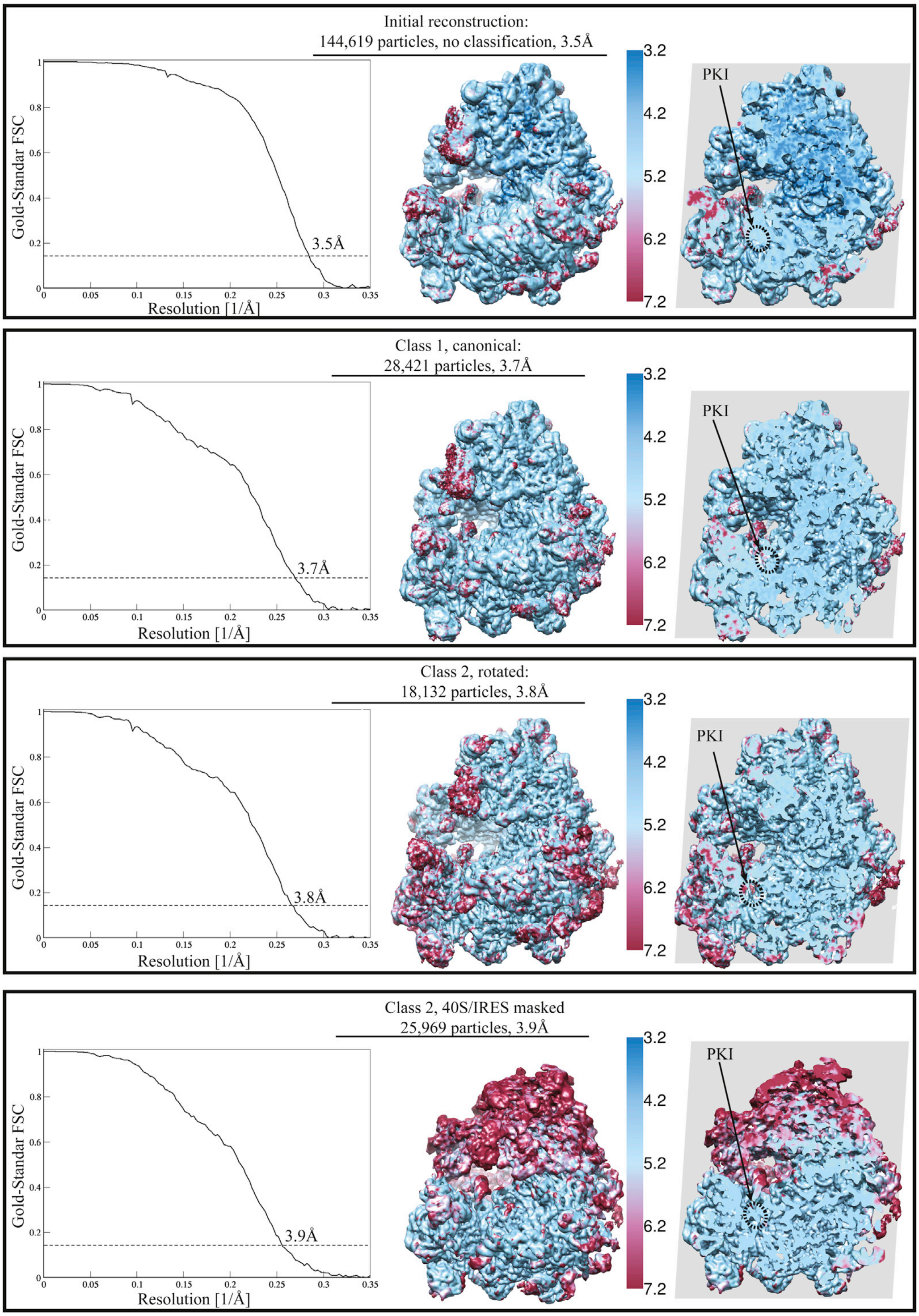

Figure S2. Resolutions for Overall Data Set and Individual Classes, Related to Figure 1

Left: Gold standard Fourier shell correlation coefficients for the reconstructions of the classes shown in Figure S1. The dashed line represents an FSC of 0.143, and the resolution at which the FSC reaches this value is shown in each case. On the right, the local resolution is shown by color, with the surface shown to the left of the color bar, and a cross-section through the ribosome on the right. Throughout much of the interior, the local resolution is significantly better than the overall resolution.

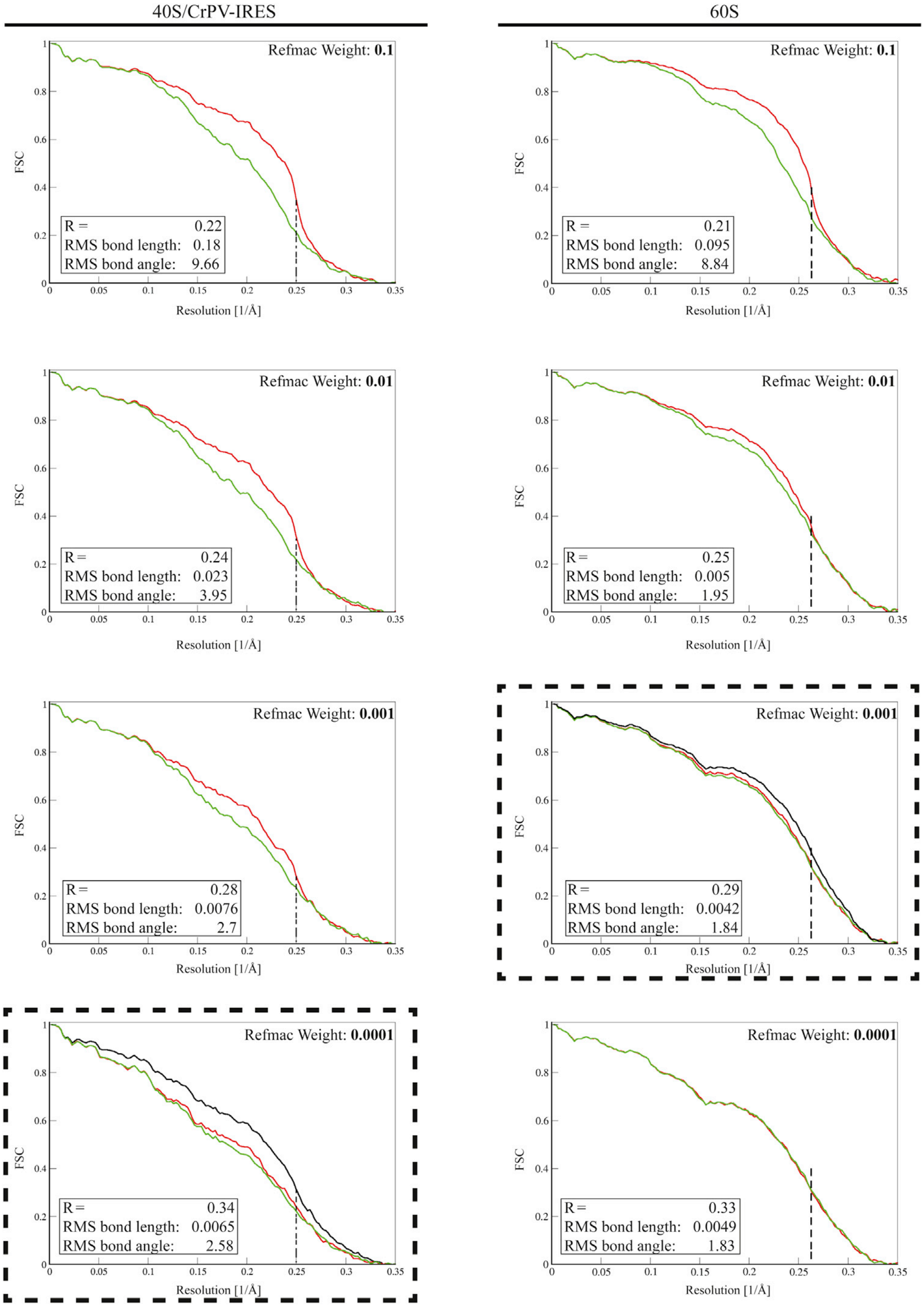

Figure S3. Model Refinement Optimization, Related to Figure 1

Use of cross-validation to optimize the weight on the experimental density in REFMAC to prevent overfitting. The weights were optimized independently for the refinements of the 40S/CrPV-IRES (left) and the 60S (right). Lower values for the Refmac weights put less weight on the geometric restraints and more weight on the experimental density. FSC_{work} curves, i.e., Fourier shell correlation curves calculated between the refined atomic model and the half-map it was refined against (see the Methods section in the main text), are shown in red. FSC_{test} curves, i.e., those calculated between the refined atomic model and the other half-map, are shown in green. Large differences between FSC_{work} and FSC_{test} are an indication of overfitting. In addition, a sharp drop in FSC_{work} at the highest resolution that was included in the refinement (indicated with vertical dashed lines) is also indicative of strong overfitting, for example as observed in the top left panel. The weighting scheme that was used for the final refinement of each subunit is shown for the curves enclosed in the dashed box. The black curve shows the FSC curve between a reconstruction from all particles and the model refined against that map.
